# Surgery

**Published:** 1866-02

**Authors:** 


### SELECTED ARTICLES.

### SURGERY.

### *Two Tumors removed from the Larynx in a Case of Aphonia of Six Years, followed by immediate Speech.* Dr. Gibb.

The patient was a single lady, of thirty-eight, who had had a bad throat for six years, the voice being reduced to a croupy whisper. During the first three years she had coughed up pieces of "flesh," one of which was an inch long and the shape of a shrimp. For the greater part of the time she could not lie down at night, from supposed cardiac disease with dyspnœa, and her complexion was very florid. On examination with the laryngoscope in April last, a long, fleshy, somewhat bulbous growth was seen to occupy the greater part of the sub-glottic space, springing from the anterior part of the larynx, below the origin of the true vocal cords, and quite immovable. The larynx in other respects was comparatively healthy, but there was much irritability and spasm of its proper muscles. After six weeks' preparation, Dr. Gibb snared a growth in the loop of wire of his laryngeal ecraseur, cut its pedicle, and withdrew it firmly held by a piece of uncut mucous membrane in the retracted loop, in a similar manner to the outer coat of the artery after ligature. He now found a second and larger growth, which had formed the bed of that already removed, and this was snared six days later in a similar manner to the first, the tumor being likewise withdrawn in the retracted wire loop of the ecraseur. The voice was immediately restored, for the mechanical obstruction was got rid of. In a few days the little wound cicatrized, no obstruction was visible, the trachea was normal, and the patient left for the North cured. The composition of the tumors was wholly epithelial cells, and to the naked eye resembled a congeries of small cysts; they were of the size of small beans. The tumors were exhibited with the wires attached to the mucous membrane, and Dr. Gibb remarked that when the loop of the wire was not violently or spasmodically drawn home, the growths invariably were withdrawn with the instrument itself. The case made, he believed, his fifteenth or sixteenth in which he had now successfully abstracted growths from the larynx by means of wire loops, and this in their entirety, not in fragments. The shape and position of the tumors in the present instance were well illustrated by a large diagram. —*Medical Circular.*

